# Editorial: New strategies in treatment of differentiated thyroid carcinoma

**DOI:** 10.3389/fendo.2022.1004734

**Published:** 2022-08-24

**Authors:** Jose Federico Carrillo, Carlos Suarez, Alvaro Sanabria, T Metin Onerci, Dhairyasheel Savant

**Affiliations:** ^1^ Head and Neck Department, National Institute of Cancerology (INCAN), Mexico City, Mexico; ^2^ Servicio de Otorrinolaringología, Hospital Universitario Central de Asturias, Oviedo, Spain; ^3^ Departamento de Cirugía, Facultad de Medicina, Universidad de Antioquia, Medellín, Colombia; ^4^ Faculty of Medicine, Hacettepe University, Ankara, Turkey; ^5^ Head and Neck Department, Asian Cancer Institute (ACI), Mumbai, India

**Keywords:** Thyroid carcinoma, treatment strategies in thyroid cancer, thyroid cancer surgery, thyroid cancer diagnosis, thyroid cancer biomarkers, radionuclide I 131, thyroid cancer radiotherapy, thyroid cancer targeted therapy

In this Research Topic of manuscripts on *New Strategies In Treatment of Thyroid Carcinoma* (TC), edited by the journal Frontiers in Endocrinology, papers have been gathered that, from the perspective of basic sciences, can potentially contribute to provide issues for a better understanding of clinical problems.

Moreover, molecular biology and studies designed to improve the intrinsic behavior of papillary carcinoma are pivotal in the development of targeted therapies. The study by Yang el al. tries to ellucidate the role of estrogen receptors on the stimulation of cancer cells, through a significant decrease on the levels of endogenous PPARγ ligands PGJ2 and 15(S)-HETE. These findings open a venue to development of endogenous ligands PGJ2 and 15(S)-HETE, to treat papillary thyroid cancer.

The role of primary cilia alterations in differentiated TC and its absence in the anaplastic variety is described, as well as its relationship to the presence of the Aurore kinase protein, which is linked to disassembly of these cilia in TC. The article by Ma et al. describes these phenomenae, as well as the possibility of other biochemical pathways implicated with these organelles like Nek1 and Plk1, with the possibility of developing drugs which could restore the physiology of primary cilia of thyroid cells like U0126 and ganetespib.

TC has increased its incidence and prevalence during the last 20 years. This is secondary to a better understanding of its presentation, as well as of diagnostic studies performed at present, from which, different image tests like high definition ultrasound, CT scan and aspiration biopsy represent the most relevant ones.

On the other hand, a significant number of reports attest to efforts to improve the diagnosis potential of the aforementioned tests. In this e-book the report by Zhang et al., establishes the modification of plasma N-glycome alteration in Thyroid Carcinoma and Benign Thyroid Neoplasms, which in the future could improve the diagnostic accuracy of ancillary tests as well as the detection of lymph node metastases and assist to design the surgical strategy of malignant lesions.

A report by Jin et al. in a large cohort of papillary carcinoma patients with Type II Diabetes Mellitus,establishes a glucose-lymphocyte ratio higher than 4.23 as well as other factors like age, tumor size and multifocality significant for the presence of central lymph node metastasis which allows discrimination of patients who could be spared a dissection of this specific compartment, with potential decrease of related complications. This reflects the present tendency to improve quality of life in TC patients already afflicted with other comorbidities. The establishment of such kind of nomograms will allow more personalized surgical strategies according to an accurate classification of risk factors (1).

In the same sense, the risk of lymph node and distant metastases in children 2-16 years of age is reported by Zeng et al. to be higher in the classic variant of papillary thyroid carcinoma, (CPTC); moreover, when associated to other risk factors like extrathyroid invasión would warrant a more aggresive treatment and closer followup than in those with ages>16 years, as well as in the presence of the follicular variant of papillary thyroid cancer.

Central lymph node metastasis (CLNM) are regarded as a predictor for local recurrence in patients with papillary thyroid carcinoma (PTC) but the role of prophylactic central lymph node dissection is controversial. Zeng et al. studied 1,054 consecutive PTC patients with the aim to identify the clinical factors associated with CLNM and develop a nomogram for making individualized clinical decisions. CLNM was determined in 31.4% (168/535) of non-Hashimoto’s thyroiditis (HT) patients versus 39.2% (83/212) in HT patients (p=0.043). They concluded, that classical PTC patients with features like male gender, age<55 years old, tumor size>1cm, and Hashimoto’s thyroiditis condition had a higher risk of CLNM.(7)

The paper by Pitalua-Cortes et al., evaluates 68Ga-PSMA-11, which is a novel tracer to detect recurrences either locorregional or metastatic in papillary thyroid carcinoma. Although a small series, with no clear definition of a gold standard, this molecular radiotracer has the potential to complement the I131 SpectCT, as well as to be of use for theragnostic application to select patients for possible therapy with Lu – PSMA–617, specifically regarding radioiodine resistant lesions.

Four papers concern different aspects related to the surgical technique used, in order to establish minimally invasive methods, the advantages of non-total thyroid resection, the attitude towards airway invasion, and the role of radiofrequency in the ablation of non- aggressive tumors. This last one alludes, consequently, to the avoidance of a surgical procedure.

At present, diagnosis of papillary microcarcinoma has increased, as already stated, and its treatment is still open to debate. However risk stratification and tuning of current systems regarding papillary thyroid cancer has increased, resulting in better design of surgical strategies in a way that patients with no lymph node metastases, multifocal tumors, and absent major extrathyroid extensión could be treated with a unilateral lobectomy as described by Zhao and Cui.

New techniques to prevent patients from neck scar and reduce complications with no inferiority results in treatment of thyroid papillary carcinoma have been introduced in recent times. The article by Rossi et al. provides a comprehensive review of the state of the art, where trans-oral endoscopic thyroidectomy with vestibular approach (TOETVA) appears as the most efficient one in terms of feasibility, expenses, and complication rate. Although requiring a learning curve experience and adequate selection of patients (carcinomas up to1-2 cm in diameter, with no lymph node metastasis, no multifocality, and with no extrathyroid extensión), along with adequate follow-up and determination of postoperative safe markers (thyroglobulin and antithyroglobulin antibody) levels, this technique is promising as long as these requirements are fullfilled, which will reduce the present oncologic concerns-including aggresive variants of TC (2, 3)- regarding safety and efficacy, which are still under discussion.

Most TC cases fall in the low to intermediate risk, however, major invasión to vascular and aerodigestive structures occurs in 7-13% of patients, and very often invasion to cricotracheal areas hinders larynx preservation. However, Piazza et al. have added perspective to endoluminal laryngotracheal invasion regarding the concept of circumpherential resection with end to end anastomosis including or not the cricoid cartilage: 25% and 75% of cases in their series, respectively. Additionally, the Shin III and Shin IV classification was present in 39 (26%), and in 62 (42%) procedures. The results regarding complication rates were 27% on the whole, with tracheostomy dependency of 4%. Limits for this technique were defined as 4 cm length in vertical extension and posterior cricoid invasion up to the aritenoids. Clearly, these procedures require extensive workup and expertise, as well as comparison with the inclusion of a total laryngectomy (4), with new investigations warranted on quality of life obtained by patients, since survival at 15 years follow-up reaches 30%.

The issue of termal energies to treat differentiated carcinoma of the thyroid is raised by Tufano et al. Radiofrequency ablation (RFA) seems, at present, the most efficient of the energies used so far. Although RFA is currently used mostly in patients with benign nodules, its indications in thyroid malignancy are in T1a and T1b tumors with no high risk factors, specifically in patients who are bad surgical candidates, refuse surgery or have a recurrent lesion in cases where previous surgery, and or radioioidine treatment make a rescue surgery technically hazardous and with potential to decrease the quality of life of the patient. Complications rates in malignancies are major (5.5%) and minor (10%) as reported in recent metaanalyses, and slightly higher (10%% and 15%) in recurrent lesions. The use of these techniques, in thyroid carcinoma, warrants performance of well designed prospective clinical trials.

Finally, Zhou et al. highlight that radiotherapy plus chemotherapy contributes to prolonged overall survival and cancer specific survival compared with radiotherapy alone in anaplastic thyroid cancer patients, regardless of surgical resection and distant metastasis, reinforcing the new concepts explored by Wang and Zaffereo and their group on targeted agents (5). In the same venue to explore the role of radiotherapy in thyroid carcinoma, recent reports on the use of upfront radiation treatment followed by rescue surgery have appeared (6), which in combination with new targeted therapies could constitute a new approach for unresectable malignancies.

An exciting area for improvement of oncologic outcomes and quality of life of TC patients is under continuous development, where biology of tumor, better diagnostic image and biologic markers tools, non invasive treatment of malignancies, and evolving surgical techniques combined with targeted therapies, as well as judicious use of external radiotherapy, contribute to solve ancillary problems and to breach old barriers in the pursue of new strategies for treatment of differentiated thyroid cancer **Figure 1**.

**Figure 1:**
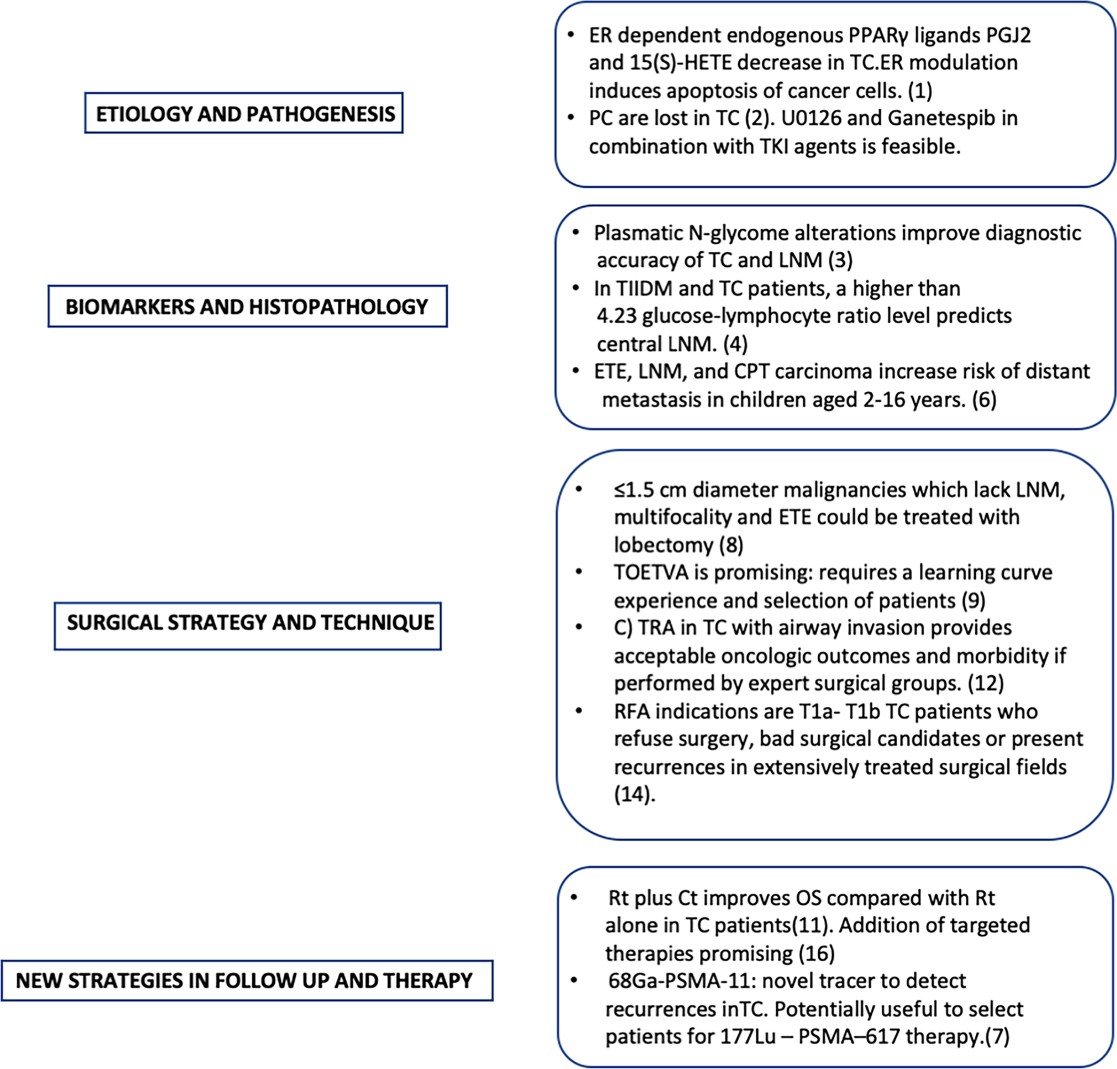
Pivotal Findings Regarding New Strategies In Treatment Of Thyroid Carcinoma: Er, Estrogen receptors; PC, Primary cilia, TC, Thyroid carcinoma; LNM, Lymph Node Metastasis; IIDM, Type II Diabetes Mellitus; ETE, Extrathyroid extension; CPT, Classic Papillary Thyroid; TOETVA, Transoral endoscopic thyroidectomy vestibular approach; (C)TRA Cricotracheal and tracheal resection with end to end anastomosis; RFA, Radiofrecuency Ablation; RT, radiotherapy; CT, Chemotherapy; OS, Overall Survival.

## Author contributions

Topic direction, analyses and criticism: JC, CS, AS. Manuscript design, writing, and drafting: JC, CS, AS, MO, DS. Manuscript review, analyses and administration: JC, CS, AS, MO, DS. All authors contributed to the article and approved the submitted version.

## Conflict of interest

The authors declare that the research was conducted in the absence of any commercial or financial relationships that could be construed as a potential conflict of interest.

## Publisher’s note

All claims expressed in this article are solely those of the authors and do not necessarily represent those of their affiliated organizations, or those of the publisher, the editors and the reviewers. Any product that may be evaluated in this article, or claim that may be made by its manufacturer, is not guaranteed or endorsed by the publisher.
